# A Survey of Psychiatric Healthcare Workers' Perception of Working Environment and Possibility to Recover Before and After the First Wave of COVID-19 in Sweden

**DOI:** 10.3389/fpsyt.2021.770955

**Published:** 2021-11-29

**Authors:** Eirini Alexiou, Steinn Steingrimsson, Magnus Akerstrom, Ingibjörg H. Jonsdottir, Linda Ahlstrom, Caterina Finizia, Helle Wijk, Alessio Degl'Innocenti

**Affiliations:** ^1^Department of Psychiatry, Forensic Psychiatric Clinic, Sahlgrenska University Hospital, Region Västra Götaland, Gothenburg, Sweden; ^2^Center for Ethics, Law, and Mental Health, Sahlgrenska Academy, University of Gothenburg, Gothenburg, Sweden; ^3^Institute of Neuroscience and Physiology, Sahlgrenska Academy, University of Gothenburg, Gothenburg, Sweden; ^4^Institute of Stress Medicine, Region Västra Götaland, Gothenburg, Sweden; ^5^School of Public Health and Community Medicine, Institute of Medicine, Sahlgrenska Academy, University of Gothenburg, Gothenburg, Sweden; ^6^Institute of Health and Care Sciences, Sahlgrenska Academy, University of Gothenburg, Gothenburg, Sweden; ^7^Department of Orthopaedics, Sahlgrenska University Hospital, Region Västra Götaland, Gothenburg, Sweden; ^8^Department of Otorhinolaryngology, Head and Neck Surgery, Institute of Clinical Sciences, Sahlgrenska Academy, University of Gothenburg, Gothenburg, Sweden; ^9^Department of Research, Development, Education and Innovation, Sahlgrenska University Hospital, Region Västra Götaland, Gothenburg, Sweden; ^10^Department of Quality Strategies, Sahlgrenska University Hospital, Region Västra Götaland, Gothenburg, Sweden; ^11^Department of Architecture and Civil Engineering, Chalmers University of Technology, Gothenburg, Sweden; ^12^Gothia Forum for Clinical Trials, Sahlgrenska University Hospital, Region Västra Götaland, Gothenburg, Sweden

**Keywords:** COVID-19 pandemic, online survey, psychiatric healthcare workers, recovery, working environment

## Abstract

**Objective:** This study aimed to investigate the impact of the first wave of the COVID-19 pandemic on perceived working environment, including the possibility to recover, among psychiatric healthcare workers (PHCWs) in comparison with pre-pandemic measures.

**Method:** A link to an anonymous, web-based COVID-19 related survey was sent *via* email to all PHCWs at a university hospital in Sweden (*n* = 1,618) in September 2020. The response rate was 38% (566 of 1,507 eligible participants). Working environment survey responses collected in 2019 were used as pre-pandemic comparators. Statistical analyses were performed to assess overall impact over time on work demands, support, motivation, and recovery, stratified by professional role, and considering variables such as access to personal protective equipment.

**Results:** The percentage of individuals responding negatively to statements about working environment increased significantly for most items after the first wave. Similarly, the increase of five of the investigated factors indicated a more negative perception of recovery during the pandemic. Registered nurses reported a greater negative impact of the pandemic on perceived working conditions and ability to recover than other professional groups. PHCWs working with patients with COVID-19 (35%) who reported being worried about becoming infected (12%) or infecting others (17%), or lacking adequate personal protective equipment (22%) were more negatively impacted regarding work environment-related items than those who did not.

**Conclusions:** PHCWs' working environment and possibility for recovery were impacted by the first wave of the COVID-19 pandemic, nurses being most affected. Although psychiatric services do not directly care for patients with severe COVID-19 infection, the results from this study suggests that mental health services should also prepare for future pandemics.

## Introduction

The psychosocial work environment among psychiatric healthcare workers (PHCWs) during the pandemic caused by severe acute respiratory syndrome coronavirus-2 (COVID-19), starting in 2020, has generally been described as being poor (1–4). Psychosocial work environment among PHCWs is important to ensure high quality and safe psychiatric care, even during extraordinary events such as pandemics.

The COVID-19 pandemic has greatly impacted healthcare workers (HCWs) both in terms of workload and mental health (5–7). Similar impacts, albeit to lesser degrees, have been seen when new strains of coronavirus have appeared in the past, such as those causing severe acute respiratory syndrome (SARS) and Middle East respiratory syndrome (MERS) (8–12). The COVID-19 pandemic has exposed HCWs to exceptional situations that can lead to increased psychological problems such as anxiety, depression, and insomnia (13–15) as well as changes in perceived work environment (16–18) as also shown by our research group (5). Related contributing factors to poor perceived working environment include excessive workload, insufficient managerial support, low possibility for repose during working hours, worries about infection, inadequate access to personal protective equipment (PPE), and over-enthusiastic media coverage (5, 19, 20). Although HCWs directly caring for patients with severe COVID-19 are at risk of experiencing negative impacts on their own mental health, those working with other patient groups such as home health care, or in other non-clinical roles have also been affected (19). For example, a recent cross-sectional study reported high prevalence of burnout, anxiety, and distress among academic otolaryngologists who were not caring for patients with COVID-19 (21).

PHCWs are an integral part of the healthcare system and despite not providing direct care for the most affected patients during the pandemic, they still provide care for patients with COVID-19 in need of psychiatric care. Furthermore, changes in work routines to decrease the risk of spreading the virus could potentially also affect PHCWs' working environment. Understanding the impact of the COVID-19 pandemic on PHCWs' working environment is imperative for facilitating planning and ensuring high quality and safe psychiatric care. The few studies available in the literature have focused on distress among PHCWs during the pandemic (2, 22, 23) but as far as we know no study investigating the self-perceived working environment during the COVID-19 pandemic among PHCWs have been published. Therefore, the aim of the present study was to compare self-perceived working environment, including job strain, support, and work engagement as well as recovery among PHCWs, before and after the first wave of the COVID-19 pandemic in Sweden. Furthermore, this study aimed to assess whether factors such as professional role, frequent worries about being infected, caring for patients with COVID-19, or having had a departmental transfer modified the rating of perceived changes in psychosocial work environment.

## Materials and Methods

### Setting

This study was conducted at a university hospital in Sweden, one of the largest university hospitals in northern Europe with a total of ~17,000 employees. The hospital provides both emergency and basic care for the 700,000 inhabitants of the Gothenburg Region and offers highly specialized care for the 1.7 million inhabitants of the region of Västra Götaland in western Sweden.

The psychiatric departments of the hospital are divided into five domains: addiction services, forensic psychiatry, general psychiatry, geriatric psychiatry, and specialized service for psychosis. In this study, all departments were considered as one psychiatric service.

### Population and Procedure

**Figure 1:**
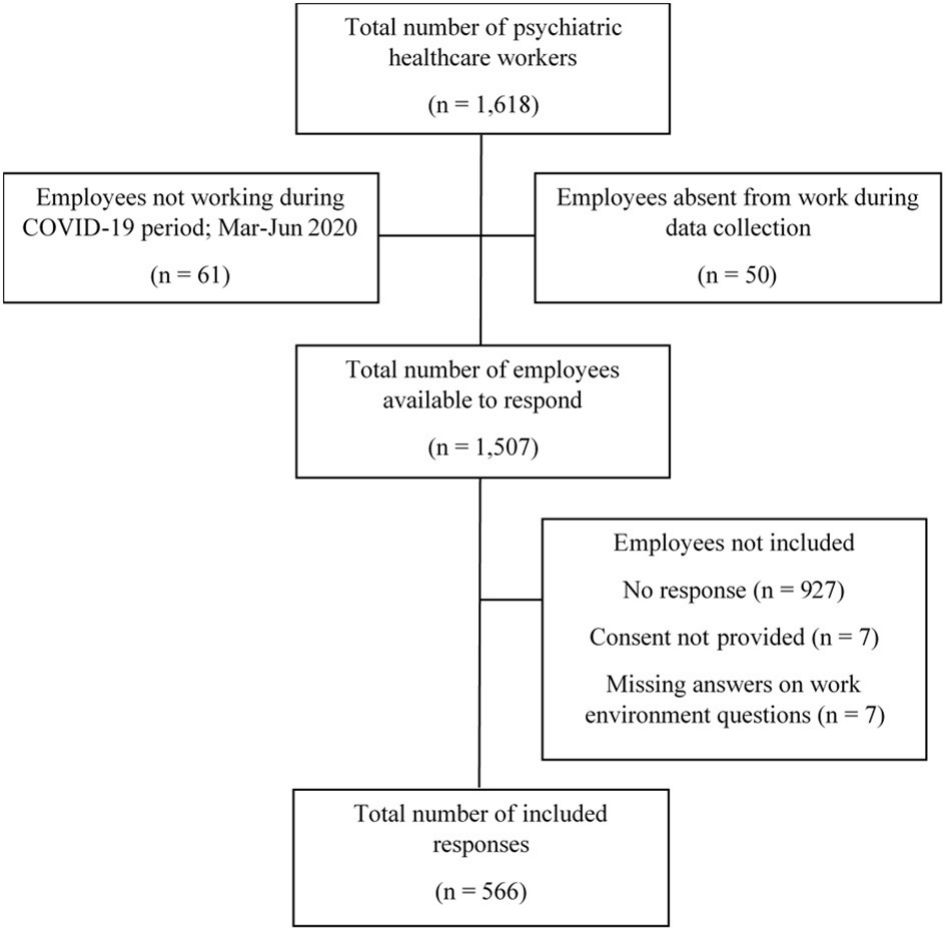
Response flowchart to online survey administered to psychiatric healthcare workers in 2020.

This study focuses on PHCWs at a university hospital in Sweden. As a part of this study, the result from a biannual work environment survey was used. The survey is based mainly on the job demands-resources (JD-R) model (24) but also includes other aspects of the organizational and psychosocial work environment. The last measure before the pandemic was during the autumn 2019. Some items from this general health surveillance survey were applied in 2020, in collaboration with the hospital's Human Resources (HR) department, as part of a web-based COVID-19-related survey. The COVID-19 related survey was produced in collaboration between researchers (expert opinion) and the operational area at the hospital (practitioners and respondents). The instrument was validated by testing it on 10 people prior to the study, to ensure that the questions were understandable. This survey was administered to all employees at the hospital, including psychiatry wards (*n* = 1,618) regardless of having direct patient contact or non-clinical work tasks. In the first week of September 2020, an invitation to anonymously participate was sent by email including a link to the survey. The possibility to answer the survey was ~5 weeks. One email reminder was provided during the last week of September 2020. After excluding employees not working (*n* = 61) or absent from work (*n* = 50) during this period, the number of eligible PHCWs for study participation was *n* = 1,507 (Figure 1). Individuals without informed consent (*n* = 7) were excluded from the study together with individuals with missing data (*n* = 7) on all 11 work environment questions, resulting in 566 post-measurement survey responses (response rate 38%) from the psychiatric departments within the hospital.

The data from the 2019 collection was used as unexposed (pre-pandemic) PHCW data in the analyses to compare to the 2020 data (exposed PHWC data). Out of a total of 1,924 as many as 1,277 PHCWs completed the survey in 2019 and the response rate was 66.4% (eligible PHCWs *n* = 1,924).

The study was approved by the Swedish Ethical Review Authority (Ref. 2020-04771) and participants provided informed consent. The study was conducted in compliance with the Declaration of Helsinki and the General Data Protection Regulation (EU) 2016/679.

### Survey and Outcome Measures

The survey was designed to be completed in 10–20 min. Demographic items including age, gender, organizational affiliation, and professional role were collected. Eleven items regarding working conditions were also included, addressing work demands, support, motivation, and recovery. These same items were included in an October 2019 employee survey, thus offering a recent measure of work conditions before the COVID-19 pandemic. All items were presented as statements with five response alternatives (strongly agree, agree, neither agree or disagree, disagree, and strongly disagree). Additional items about work placement during the pandemic, worries about getting infected with the novel COVID-19, and access to PPE were included. When answering questions about work conditions, participants were asked to think back to how they perceived the situation during the intensive period of the pandemic during spring 2020 (5).

### Statistical Analyses

The Shapiro–Wilk-test and visual inspection of histograms were used to test the work environment measures for normality. An assumption of normality was judged to be plausible and parametric methods on untransformed data were used in the analyses. Statistical significance was set at *p* < 0.05, and two-sided confidence intervals were used.

The impact of the COVID-19 pandemic on the psychosocial working environment was assessed in three steps: (1) the overall impact of the pandemic was assessed using the pre-pandemic and post-first-wave responses, (2) differences in the impact between professional roles were investigated, and (3) effect modifiers explaining the potential variation between different groups (frequent worries about being infected, caring for patients with COVID-19, or having had a departmental transfer) were investigated using post-first-wave responses.

An overall impact was investigated both for the average survey score as well as for the percentage respondents reporting a negative response (strongly disagree or disagree), as average levels may mask changes between the response categories within a group.

In the first step, mixed-effects models (Proc Mixed in SAS version 9.4; SAS Institute, North Carolina, USA) were used to assess the overall impact of the pandemic with time (2019 or 2020, nested within departments) as a fixed effect and information on departments as random effects. Hypothesis testing for fixed effects was performed using Wald tests, and tests of random effects were performed using likelihood ratio tests (25).

In the second step, differences in the impact of the pandemic between professional roles (physicians, registered nurses, assistant nurses, administrative staff, and other occupations) were investigated either by adding interaction terms between the time variable and the variable for professional roles, or by stratifying the analyses according to the above.

In the third and final step, the effect on the working conditions of working with patients with COVID-19 (yes or no), being transferred to another department (never, occasionally, and most of the time), having a strong worry of becoming infected (never, rarely, occasionally, daily, and many times each day) and having access to enough PPE while working with patients with COVID-19 (often or very often, occasionally, rarely, or very rarely), were investigated using mixed-effects models with the effect modifiers added as fixed effects and information on the departments as random effects. For these analyses, five items were selected from the survey representing perceived working conditions such as job demands (one item covering quantitative demands), job resources (two items covering competence and support), motivation (one item) and possibility for recovery (one item).

## Results

In total, 566 PHWCs responded (38%; eligible PHWCs *n* = 1,618) to the survey, a majority being female (75%). The most common professions were registered nurses or nurse assistants (19 and 28%, respectively) and age categories were mostly equally distributed (Table 1). The distribution of age and professions were similar between the two surveys. There was a slight difference in the gender distribution with a somewhat larger proportion of women in 2019 but there were a large proportion of missing answers regarding the respondents' gender in 2019 (7%) which could partly explain this (Table 1). In total, 35% (*n* = 197) of the PHCWs participating in the survey reported themselves as having taken care of patients with COVID-19. As shown in Table 1, most of the PHCWs stayed at their regular workplace during wave 1 (83%, *n* = 470). Regarding worries about infections among PHCWs, 12% (*n* = 69) reported feeling very worried about becoming infected with COVID-19. Finally, only 19% (*n* = 106) of responding PHCWs reported that they always/very often had access to PPE and 11% (*n* = 64) quite often.

**Table 1:** Demographics and other characteristics of psychiatric healthcare workers answering a workplace survey in September 2020 and October 2019 [presented as *n* (%)].

		**2020**	**2019**
**Variable**	**Category**	***n*** **= 566**	***n*** **= 1,277**
Occupational status	Physician	49 (9)	141 (11)
	Registered nurse	110 (19)	283 (22)
	Assistant nurse	162 (28)	321 (25)
	Administrator	58 (10)	112 (9)
	Other	187 (33)	420 (33)
Age (years)	≤ 29	36 (6)	127 (10)
	30–39	142 (25)	342 (28)
	40–49	123 (22)	280 (23)
	50–59	149 (26)	290 (23)
	≥60	115 (20)	201 (16)
Gender	Female	422 (75)	842 (70)
	Male	138 (24)	342 (29)
	Other	3 (1)	9 (1)
Caring for patients with COVID-19	Yes	197 (35)	
	No	369 (65)	
Hospital department	Regular department	470 (83)	
	Various departments	41 (7)	
	A different department	25 (4)	
	Other	30 (5)	
Very worried about being infected	Many times a day	69 (12)	
	Everyday	98 (17)	
	Sometimes	118 (21)	
	Once in a while	182 (32)	
	Never	91 (16)	
Access to personal protective equipment	Very often or always	106 (19)	
	Quite often	64 (11)	
	Sometimes	39 (7)	
	Quite rarely	22 (4)	
	Very rarely or never	27 (5)	
Have not been in contact with COVID-19 patients		305 (54)	

### Overall Effects of the COVID-19 Pandemic on Working Conditions and Recovery

**Figure 2:**
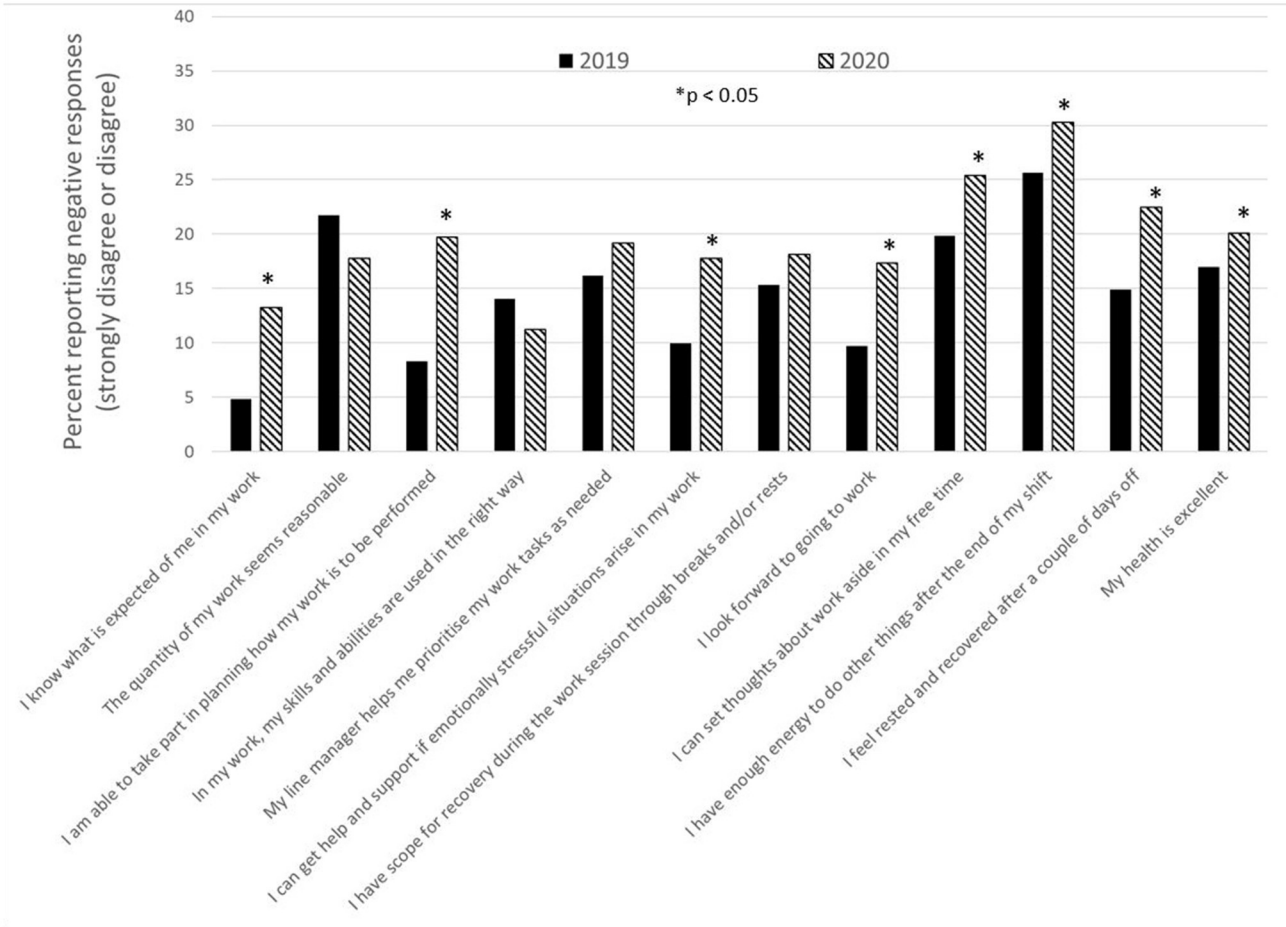
The percentage of negative responses (strongly disagree or disagree) to statements regarding work environment and possibility for recovery before and after the first wave of the COVID-19 pandemic.

The percentages of PHCWs reporting a negative working situation measured either before or after the first wave of the pandemic, i.e., strongly disagreed or disagreed with the different statements in the survey regarding work environment and recovery, are presented in Figure 2. The percentage of negative responses increased significantly (*p* < 0.05) from 2019 to 2020 for 8 of the 12 items, thereby indicating a deterioration regarding the perceived working environment situation among the study population during the COVID-19 pandemic.

Overall effects of the COVID-19 pandemic on working conditions and possibility for recovery compared to the situation before the pandemic are presented in Table 2. For 5 of the 11 investigated factors, a significant decrease was observed in 2020 compared to survey results from 2019, thus indicating a more negative perception of work environment and recovery during the pandemic. However, for 1 of the 11 investigated factors (quantitative demands, *p* = 0.01) a more positive perception of the work environment was reported after the pandemic's first wave compared to pre-pandemic results.

**Table 2:** Overall effects of the COVID-19 pandemic on working conditions and possibility for recovery compared to the situation before the pandemic.

**Survey item**	**Estimate (95% CI)**	***p*** **-value**
I know what is expected of me in my work	−0.35 (−0.47 to −0.22)	<0.001
The quantity of my work seems reasonable	0.26 (0.06 to 0.46)	0.01
I am able to take part in planning how my work is to be performed	−0.40 (−0.59 to −0.20)	<0.001
In my work, my skills and abilities are used in the right way	0.13 (−0.04 to 0.29)	0.1
My line manager helps me prioritize my work tasks as needed	−0.17 (−0.42 to 0.08)	0.2
I can get help and support if emotionally stressful situations arise in my work	−0.38 (−0.57 to −0.20)	<0.001
I have scope for recovery during the work session through breaks and/or rests	0.07 (−0.16 to 0.31)	0.5
I look forward to going to work	−0.29 (−0.45 to −0.13)	<0.001
I can set thoughts about work aside in my free time	−0.15 (−0.32 to 0.01)	0.07
I have enough energy to do other things after the end of my shift	−0.06 (−0.25 to 0.13)	0.6
I feel rested and recovered after a couple of days off	−0.20 (−0.38 to −0.01)	0.04
In general, would you say your health is^**^	0.06 (−0.05 to 0.18)	0.3

** *Scale is reversed, less is better*.

### Differences in the Overall Effect of the COVID-19 Pandemic on Perceived Working Conditions and Recovery Between Professional Groups

**Figure 3:**
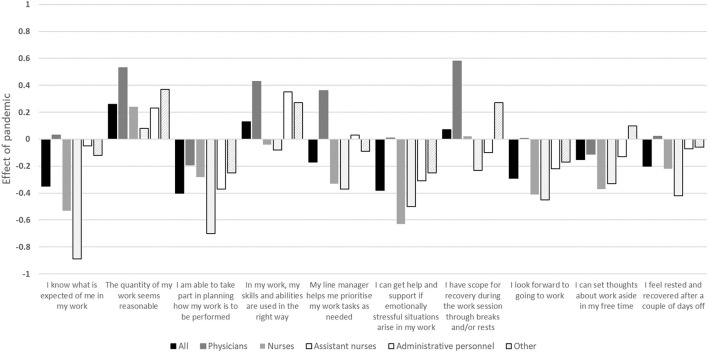
The overall effects of the COVID-19 pandemic on working conditions and recovery stratified for occupational status compared to the situation before the pandemic i.e., a negative result indicates a more negative perception of work environment and recovery during the pandemic, and vice versa. The overall effect size should be related to the item's response scale ranging between 1 (strongly disagree) and 5 (strongly agree).

Figure 3 illustrates the overall effects of the COVID-19 pandemic on self-reported working conditions and recovery, stratified by professional role and compared to the pre-pandemic situation, i.e., a negative result indicates a more negative perception of work environment and recovery during the pandemic, and vice versa.

A significant difference (*p* < 0.05) was observed between the professional groups for all items except for quantitative demands (*p* = 0.06) and feeling rested and recovered after a couple of days off (*p* = 0.1). In general, physicians were affected to a lesser degree compared to the other professional roles, especially when compared to registered and assistant nurses (Figure 3).

### Effect Modifiers Explaining the Potential Variation Between Different Groups

**Figure 4:**
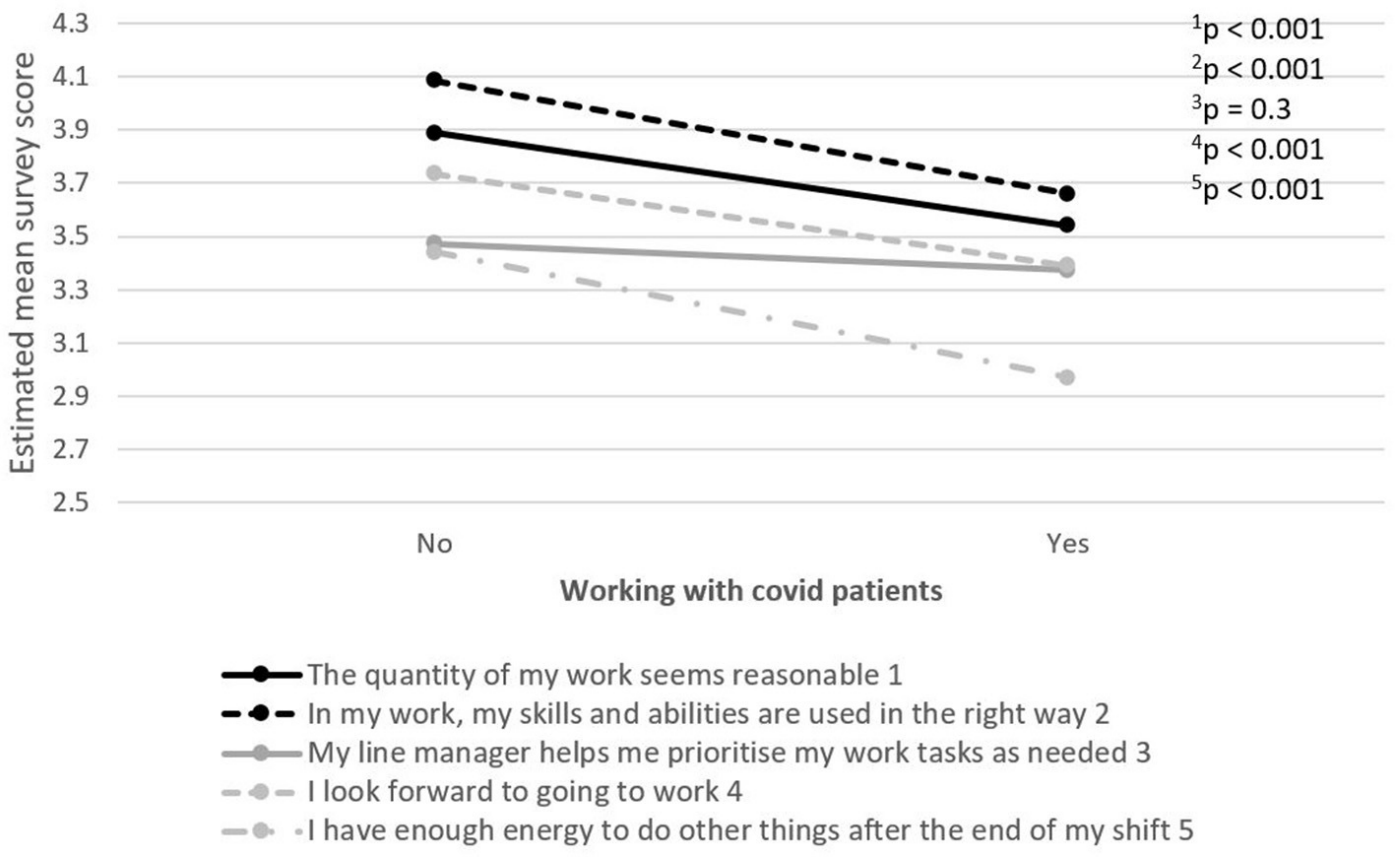
The relationship between five selected work environment items in terms of job demands and resources, motivation and possibility for recovery, and working with patients with COVID-19. The item's survey score ranges between 1 (strongly disagree) and 5 (strongly agree).

**Figure 5:**
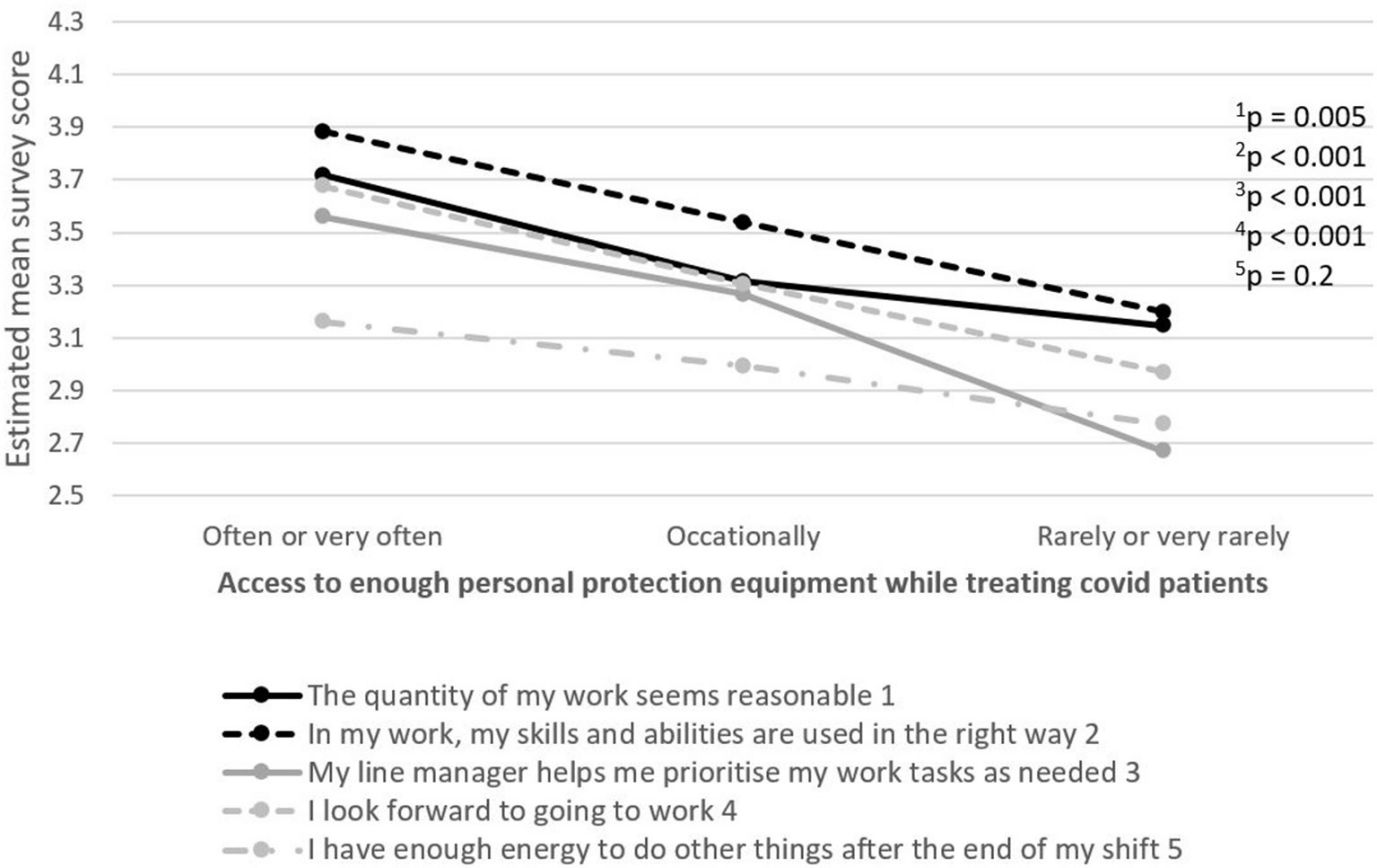
The relationship between five selected work environment items in terms of job demands and resources, motivation and possibility for recovery, and lacking access to enough personal protection equipment while working with patients with COVID-19. The item's survey score ranges between 1 (strongly disagree) and 5 (strongly agree).

The relationship between five selected work environment items in terms of job demands and resources, motivation and recovery, and factors of potential importance for how work environment and recovery is perceived, were examined. PHCWs working with patients with COVID-19 or lacking sufficient access to PPE while working with patients with COVID-19 reported a more negative perception of the working environment on most items, compared to PHCWs not working with patients with COVID-19 or not lacking sufficient PPE access, respectively (Figures 4, 5).

**Figure 6:**
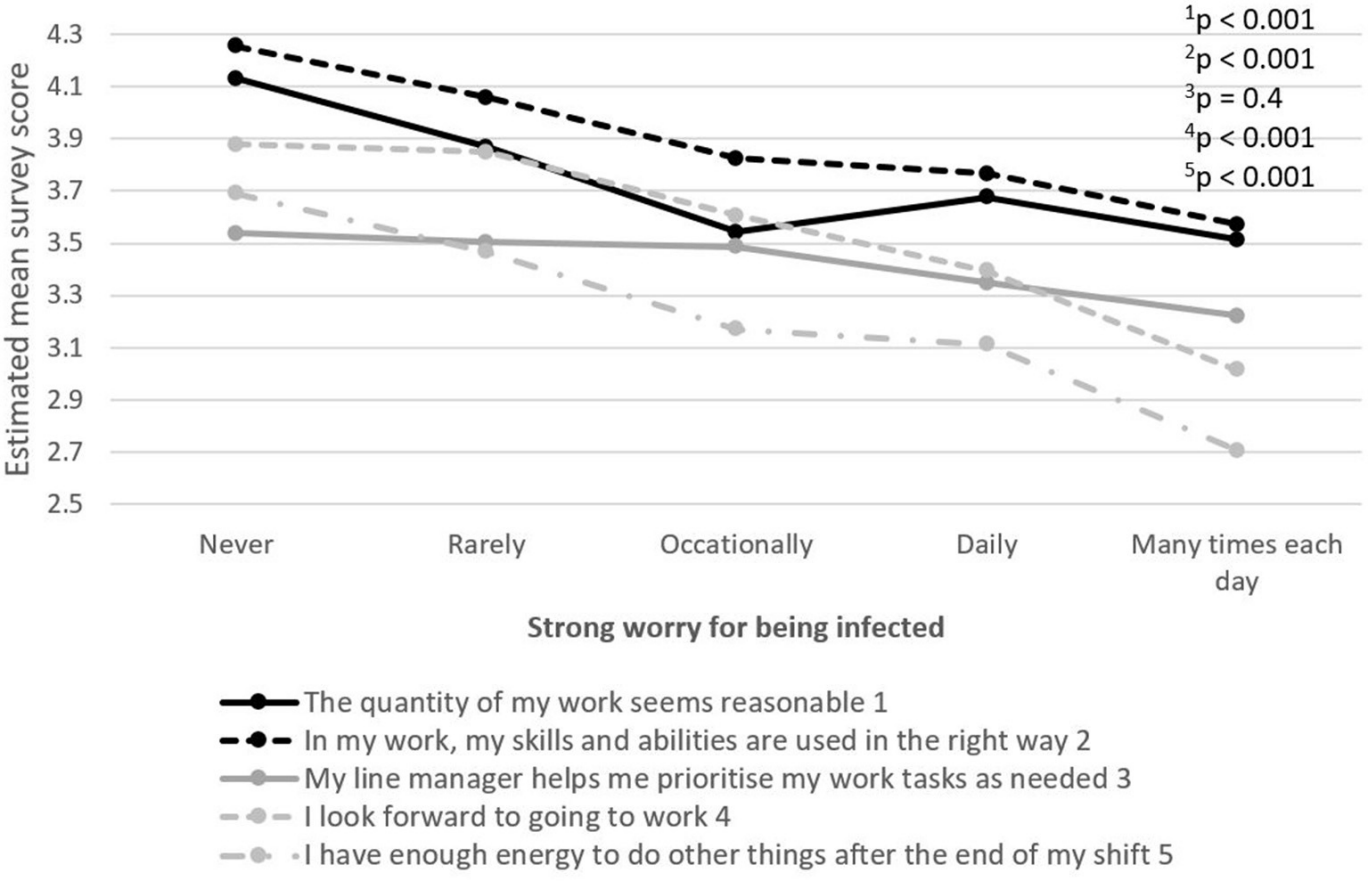
The relationship between five selected work environment items in terms of job demands and resources, motivation and possibility for recovery, and strong worry of becoming infected with COVID-19. The item's survey score ranges between 1 (strongly disagree) and 5 (strongly agree).

Similar results were seen among those who had a strong worry of being infected with COVID-19 (Figure 6).

## Discussion

The aim of the present study was to examine the impact of the first wave of the COVID-19 pandemic on PHCWs working in Sweden. Specifically, we wanted to examine whether changes made in healthcare services due to the pandemic affected PHCWs' perceptions of their own working environment and possibility for recovery.

Major findings of this study include a negative impact of the pandemic on perceived working environment and possibility for recovery compared to the pre-pandemic situation for PHCWs. This was expressed in terms of self-reported lower work motivation and lower possibility for recovery. Furthermore, PHCWs reported facing difficulties in planning their work and in knowing what was expected of them in the workplace during the first wave of the pandemic. As for help and support, PHCWs assessed their possibility to get help and support when emotionally stressful situations occurred at work as lower during the first wave of the pandemic compared to the pre-pandemic situation. This is in line with what HCWs in general have reported (5) but highlights that the working environment of psychiatry departments have also been highly influenced during the COVID-19 pandemic. There are several plausible reasons for the observed effects on work environment within psychiatry. Regardless of workplace, most people have been affected by the covid-19 pandemic, thus poorer outcome regarding work environment could generally been expected in all workplaces but perhaps more within hospital environment were people had to go to work despite the situation. The effects on the general population could plausibly also influence patients with mental health illness causing more pressure on psychiatric departments. Even though psychiatric departments are not classified as front-line regarding caring for covid-patients, they are affected since around one third of the employees have been in contact with COVID-19 patients. One risk may be that access to equipment and information is prioritizes among front-line departments such as intensive care, leaving non-frontline departments such as psychiatry with less preparation, information and support. Handling patients with infections, is indeed far from routine care in these departments. The general effect on the hospital has plausibly also affect the management as well as ordinary support, such as HR and administration even in psychiatric departments giving that large part of the hospital needed to prioritize differently during the pandemic.

Another important finding of this study was that PHCWs reported different experiences of the working environment and recovery depending on their professional role. Physicians and administrative personnel were to a lesser degree affected during the first wave of the pandemic compared to registered nurses and assistant nurses. This is in line with another study indicating increased anxiety scores reported by nurses in general compared with other health professionals during the COVID-19 pandemic (24, 26). A possible explanation for this could be that nurses have a more direct contact with patients compared to physicians and administrative personnel (27, 28). However, a potential bias to this finding could be who of the physicians and administrative personnel that answered the survey.

Furthermore, in this study, we see that exposure to patients with COVID-19 is related to higher level of negatively perceived working environment and recovery among PHCWs during the first wave of the pandemic. This was seen among PHCWs who were transferred to COVID-19-specific departments of psychiatric patients with COVID-19 symptoms. PHCWs caring for patients with COVID-19 expressed greater difficulties coping with the work burden as well as with finding energy for activities outside of work than those who did not care for such patients. They also reported feeling that their skills and abilities were not utilized to their furthest extent, which indicates that PHCWs assigned to care for patients with COVID-19 need specific support, as working in this specialty area of care requires skills, experience and practice that PHCWs could be lacking. Taken together, support in the workplace needs to be adapted to the different professional roles and skillsets during pandemics or other extraordinary events.

Furthermore, in this study, we see that exposure to patients with COVID-19 is related to higher reported level of negative responses to perceived working environment and recovery among PHCWs during the first wave of the pandemic. This was possible to observe among PHCWs who were transferred to COVID-19-specific departments of psychiatric patients with COVID-19 symptoms. PHCWs caring for patients with COVID-19 expressed greater difficulties coping with the work burden as well as with finding energy for activities outside of work than those who did not care for such patients. They also reported feeling that their skills and abilities were not utilized to their furthest extent, which indicates that PHCWs assigned to care for patients with COVID-19 need specific support, as working in this specialty area of care requires skills, experience and practice that PHCWs are often lacking.

Similar perceptions of the working environment and recovery were reported by PHCWs experiencing a strong worry of becoming infected during the COVID-19 pandemic. In a study conducted by (29), HCWs who worried about their own health and who had friends or relatives who tested positive for COVID-19, had a higher probability of experiencing anxiety and depression than those who did not. Being worried about the health consequences of the disease and about the possibility of infecting family and friends were identified as the most frequent concerns of HCWs during the H1N1 influenza outbreak (30). The same concern among HCWs is prevailing during the current COVID-19 pandemic and might be especially true for staff living with people considered to belong to a risk group. Thus, taking concerns of infection seriously at the workplace will also improve the working conditions among PHCWs during a pandemic.

Lastly, lacking access to appropriate PPE during the COVID-19 pandemic was also reported among PHCWs with a lower perception of working environment and possibility for recovery and echoes our previous study (5) including HCWs in general. A decreased feeling of safety among HCWs due to PPE shortages is common (31, 32). When combined with concerns of spreading the virus to others, caring for patients with COVID-19 while having limited/insufficient access to PPE makes it difficult for HCWs to assess their own risk of exposure to the novel coronavirus at work. Without proper PPE, HCWs treating patients with COVID-19 are more likely to become ill and to develop a fear of spreading the virus to family members and friends (33).

### Limitations and Strengths

One study limitation is the relatively low survey response rate from PHCWs, something that was in-line with similar studies conducted during the same period (5, 34–36). This may be partly explained by the idea that some employees who did not work directly with COVID-19 patients perhaps did not believe that the survey was aimed toward them, and thus refrained from responding. Another potential explanation was the dilemma of asking employees to answer a survey during a period of high workload, though a strength is that the pandemic data collection was conducted during a relatively calm period between the first and second wave, which started around November 2020 in Sweden. This increased the possibility that respondents could reflect over their working situation without simultaneously having their highest workload caring for COVID-19 patients. A potential bias is also that the response rate for the physicians and administrative personnel does not reflect the full picture on how these professions are affected. This survey was considered to be important and maybe the only way to improve knowledge about perception of working environment. The major strength of this study is that a pre-pandemic measure of working environment conditions was available for comparison. It should be noted that data were collected on a departmental level and thus individual data cannot be followed over time, something we hope being able to apply in future studies. The identification of any resilience or protective factors among the study participants is another important aspect that needs to be addressed in the future.

## Conclusions

This study shows that PHCWs' perceived working environment and possibility for recovery have been impacted by the first wave of the COVID-19 pandemic. The impact differed depending on professional role, with registered nurses and assistant nurses being more negatively affected by the pandemic than for example physicians. Although psychiatric services do not directly care for patients with severe COVID-19 infection, this study suggests that mental health services should also be prepared for future pandemics and other global catastrophes.

## Data Availability Statement

The raw data supporting the conclusions of this article will be made available by the authors, without undue reservation.

## Ethics Statement

The studies involving human participants were reviewed and approved by the Swedish Ethical Review Authority (Ref. 2020-04771). The patients/participants provided their written informed consent to participate in this study.

## Author Contributions

EA designed the study, collected and analyzed the data, and drafted and revised the manuscript. SS designed the study, analyzed the data, and drafted and revised the manuscript. MA made the statistical analyses, analyzed the data, and revised the manuscript. IJ, LA, CF, HW, and AD'I designed the study, collected and analyzed the data, and revised the manuscript. All authors contributed to the article and approved the submitted version.

## Conflict of Interest

The authors declare that the research was conducted in the absence of any commercial or financial relationships that could be construed as a potential conflict of interest.

## Publisher's Note

All claims expressed in this article are solely those of the authors and do not necessarily represent those of their affiliated organizations, or those of the publisher, the editors and the reviewers. Any product that may be evaluated in this article, or claim that may be made by its manufacturer, is not guaranteed or endorsed by the publisher.
